# Pathway-controlled fast-track rehabilitation after total knee arthroplasty: a randomized prospective clinical study evaluating the recovery pattern, drug consumption, and length of stay

**DOI:** 10.1007/s00402-012-1528-1

**Published:** 2012-05-27

**Authors:** Adrianus den Hertog, Kerstin Gliesche, Jürgen Timm, Bernd Mühlbauer, Sylvia Zebrowski

**Affiliations:** 1 Am Fuhrenkamp 2, 27798 Hude, Germany; 2 Malteser Krankenhaus St. Johannes-Stift, Johannisstraße 21, 47198 Duisburg-Homberg, Germany; 3 Department of Mathematics and Computer Science, University of Bremen, Achterstrasse 30, 28359 Bremen, Germany; 4 Institute for Pharmacology, Klinikum Bremen-Mitte GmbH, St.-Jürgen-Str. 1, 28177 Bremen, Germany; 5 Paracelsusklinik Bremen, in der Vahr 65, 28329 Bremen, Germany

**Keywords:** AKSS score, Fast-track rehabilitation, Controlled pathway, Total knee arthroplasty, TKA, WOMAC score

## Abstract

**Purpose:**

To investigate *fast*-*track* rehabilitation concept in terms of a measurable effect on the early recovery after total knee arthroplasty (TKA).

**Methods:**

This was an open, randomized, prospective clinical study, comparing the *fast-track rehabilitation*—a pathway-controlled early recovery program (Joint Care^®^)—with *standard* postoperative rehabilitation care, after TKA. Overall, 147 patients had TKA (*N* = 74 *fast-track rehabilitation,* *N* = 73 *standard* *rehabilitation*). The *fast-track rehabilitation* patients received a group therapy, early mobilization (same day as surgery) and 1:1 physiotherapy (2 h/day). Patient monitoring occurred over 3 months (1 pre- and 4 post-operative visits). The *standard rehabilitation* group received individual postoperative care according to the existing protocol, with 1:1 physiotherapy (1 h/day). The cumulative American Knee Society Score (AKSS) was the primary evaluation variable, used to detect changes in joint function and perception of pain. The secondary evaluation variables were WOMAC index score, analgesic drug consumption, length of stay (LOS), and safety.

**Results:**

After TKA, patients in the *fast-track rehabilitation* group showed enhanced recovery compared with the *standard* *rehabilitation* group, as based on the differences between the groups for the cumulative AKSS (*p* = 0.0003), WOMAC index score (<0.0001), reduced intake of concomitant analgesic drugs, reduced LOS (6.75 vs. 13.20 days, *p* < 0001), and lower number of adverse events.

**Conclusion:**

For TKA, implementation of pathway-controlled *fast-track* rehabilitation is achievable and beneficial as based on the AKSS and WOMAC score, reduced intake of analgesic drugs, and reduced LOS.

## Introduction

Total knee arthroplasty (TKA) is a standard procedure in orthopedic surgery [1, 2]. The incidence of TKA in the western countries is 150–200/100,000 inhabitants [3, 4]. The number of surgeries worldwide is increasing annually whilst the length of stay (LOS) in the hospital is decreasing [3, 5]. Nevertheless, after a TKA, the LOS in a hospital or rehabilitation environment varies from a mean of 35 days (Japan) [6], 13.9–14.3 days (Germany) [7, 8], 7.6 days (Scotland) [9], 3–4 days in specialized hospital units (Denmark) [10, 11], and even same-day discharge [12]. It appears that the LOS is not only dependent on the clinical outcome, but is also influenced by logistical factors at the treatment center, the patient’s clinical features, as well as traditions and cultural factors (urban or rural living environment) and personal factors (co-morbidities, social and marital status) [6, 13, 14]. Moreover, the national health reimbursement policies may also influence the LOS after TKA. For example in Germany, the health insurances reimbursement for hospitals, based on the Diagnose Related Group, defines a minimum and maximum LOS for a TKA that is accompanied by a fixed budget per patient, with both items being revised annually. For a TKA in Germany, the defined minimum LOS in 2005 was 6 days, in 2007 was 5 days, and in 2010, 4 days [7, 15].

Over the last decade, the concept of clinical pathways has been introduced in various specialized clinical and applied to surgical procedures, including TKA. The aim of clinical pathways is to use streamlined procedures and protocols to improve medical the quality of the treatment, minimize unnecessary variation in care, and reduce costs [16–22]. Protocols for clinical pathways coordinate the activities of multifunctional teams (including physicians, nurses, physiotherapists) involved in providing care for patients with a particular diagnosis or required procedure. Clinical pathways are typically procedure and hospital specific; they are developed by a specialized care team to create an optimal regimen of patient-centered care that is tailored to a specific institution [23–26]. Clinical pathways have a strong influence on both the medical outcome and the LOS [27].

Because of the increased awareness that a successful TKA and shorter LOS are achievable, health-care professionals in countries including Germany are interested in clinical pathways with *fast-track* approaches such as those reported from Denmark [10, 17, 28–30]. Postoperative rehabilitation with a focus on early mobilization is an accepted influencing factor in TKA and is part of a safe and enhanced care concept [31]. Early mobilization likely reduces the risks of thrombosis, pneumonia, bladder infection, although prospective clinical studies specifically investigating these aspects are still lacking [17]. In addition, there is little data available regarding the impact of *early* rehabilitation care after TKA on the recovery pattern as a medical outcome parameter [14, 32] and on prospective studies in this area of orthopedic surgery [17, 24]. Thus, the aim of our prospective, randomized, comparative clinical study presented here was to evaluate the effect of *fast-track* rehabilitation concept on the early recovery pattern after TKA and to assess its implementation in an orthopedic center in Germany.

## Patients and methods

### Ethics

The study was conducted according to the World Medical Association’s Declaration of Helsinki (1964, version 2005) [33] and to Good Clinical Practice (GCP). Approval was obtained by the state medical board of Lower Saxony, Germany. All patients were informed about the details of the study and provided a signed informed consent to participate in the study.

Case calculation, randomization, and monitoring of the study were performed through an independent institute (Department of Mathematics and Computer Science, University of Bremen, Bremen, Germany). All medical and paramedical professionals participating in the study were trained regarding the study design; ethical, legal, and scientific standards in clinical trials, using the principles of GCP. The patient demographic characteristics are summarized in Table 1.

**Table 1:** Demographic characteristics

Parameter	Control group standard care rehabilitation	Joint Care^®^ group fast-track rehabilitation	Fisher *p* value
Number of patients (*N*)	73	74	
Male *N* (%)	20 (27.40 %)	23 (31.08 %)	
Female *N* (%)	53 (72.60 %)	51 (68.92 %)	
Age (years), Mean ± SD	68.25 ± 7.91	66.58 ± 8.21	
BMI (kg/m^2^), Mean ± SD	30.38 ± 6.05	31.17 ± 5.82	
Height (cm)	167.0	167.4	
Diagnoses for surgery (*N*)			
Degenerative arthritis	72	72	
Posttraumatic arthritis	1	0	
Ahlbäck’s disease	0	2	
Surgery on left/right knee (*N*)	37/36	36/38	
Arthritis in the contralateral knee (without surgical procedure) (*N*)	34	38	
Secondary disorders or concomitant diseases			
Cardiac co-morbity	39 (53 %)	50 (67 %)	0.09
Gastrointestinal	14 (19 %)	16 (22 %)	0.8
Allergies	5 (7 %)	4 (5 %)	0.7
Kidney/urinary tract	4 (5%)	2 (3%)	0.4

Summary of the demographic characteristics for patients enrolled into the control rehabilitation study group or the fast-track rehabilitation study group. All patients had elective TKA and most of the patients were treated for degenerative arthritis

*BMI* body mass index, *SD* standard deviation, *N* number

### Study design

This was an investigator-initiated study, with a prospective, open, randomized, case control design. The study consisted of a *standard rehabilitation* group and *fast-track rehabilitation* group. Patient monitoring was scheduled for 5 visits (V) over 3 months as follows: V0 the day prior to surgery, V1: 5–7 days, V2: 15–23 days, V3: 6 weeks, V4: 3 months postoperatively.

### Inclusion criteria

The inclusion criteria were male and female patients (age range 40–85 years), admitted for elective TKA.

### Exclusion criteria

The exclusion criteria were missing informed consent, lack of cooperation capability, American Society of Anesthesiologists (ASA) score >3, rheumatoid arthritis, cancer co-morbidity, alcohol or drug abuse, previous major surgery on the affected joint, neurologic or psychiatric disease, pregnancy, and participation in other clinical studies.

### Total knee arthroplasty

This investigation took place in a non academic hospital specializing in orthopedic surgery in north-west Germany, dealing with regional patient population. About 3,000 surgeries per year (including day surgery) are performed at the hospital; of these, TKA accounts for about 300 orthopedic surgeries per year.

For the planned TKA, preoperative data were collected on the day prior to the surgery. For all patients in the study, one specialized surgical team performed the TKA (one surgeon, two anesthetists, and a team of five operating room nurses). All patients were treated with the same surgical technique, using tourniquet 350 mmHg, subvastus approach, no drains, cemented fixation, and the same implant (AGC Knee, Biomet Inc. Warsaw, Indiana USA). All patients received combined spinal analgesia during the procedure with bupivacaine 0.5 % and patient-controlled epidural analgesia with a solution of ropivacaine 0.15 %, fentanyl 0.1 %, and clonidine 0.02 % in NaCl for 48 h postoperatively.

### Discharge criteria

Discharge occurred only if the preset criteria were met. The discharge criteria for both study groups were: patient feels comfortable; low to moderate pain (indicating adequate analgesic medication); no wound leakage; independence in ‘activities of daily living’ (ADL) such as independent transfer, body hygiene, etc.; Independent mobility (partial weight bearing, walking distance >250 m). Discharge criteria were examined by the nursing team and authorized by the surgeon.

### Standard postoperative rehabilitation

In the *standard rehabilitation* group, patients received standard postoperative care according to the existing protocols on an individual care basis according to patient’s subjective demands (internal documents, Stenum Hospital; Department for Knee Surgery, Ganderkesee, Germany). The standard postoperative rehabilitations protocol includes intravenous fluid program for the first 24 h after surgery; first mobilization on the second day after surgery, daily physiotherapy in single exercises (1 h): walking exercises, passive flexion–extension of the knee up to 90-00-00°, strengthening of the lower limb muscles, respiratory training. The types of exercises used for the *standard rehabilitation* group are similar to those used for the *fast-track rehabilitation* group. The differences in the physiotherapy between the two study groups were mainly in the timing after the surgery when the physiotherapy started and the duration of the physiotherapy sessions.

Patients in the *standard rehabilitation* group were accommodated in three-bed hospital units. Individual pain medication, and discharge planning when the patient felt fit for it were performed according to the discharge criteria, specified above. Patients in the *standard rehabilitation* study group were not informed about the intended length of stay.

### Fast-track postoperative rehabilitation

The *fast-track* *rehabilitation* program used was Joint Care^®^ (Biomet Europe BV, The Netherlands). The program is characterized by patient-focused care and early mobilization with standardized postoperative milestones; these include getting up on the day of the surgery, climbing stairs 2 days after surgery, improved logistical organization involving a case manager, saying positive messages to the patient *‘yes, you can’*, and using *competitive care* by comparing the progress with fellow patients.

During the current study, patient in the *fast-track rehabilitation* group received class-type, group therapy on the same day as the TKA surgery. Patients were accommodated in a three-bed hospital units, received early mobilization (starting on the day of the surgery), standard intensive physiotherapy (2 h daily) with focus on ADL in a living room environment, and individual case management. Patients knew that early discharge was scheduled for the postoperative day 6. Nevertheless, the discharge criteria had to be fulfilled; if this was not the case, discharge was postponed.

### Post-discharge treatment

After the discharge, all patients from both study groups received the same daily exercise program, for duration of 18 days in a single rehabilitation center (Rehaklinik am Meer, Bad Zwischenahn, Germany).

### Safety

Patient safety was monitored throughout the study as the occurrence of adverse events (AE). These were classified according to severity: ‘severe’, ‘minor’, and according to relatedness to the surgical procedure: ‘very likely’, ‘likely’, ‘unlikely’, or ‘not related’.

### Data evaluation

Data were evaluated using the American Knee Society Score (AKSS) [34] and the WOMAC osteoarthritis index [35, 36]; both score instruments are widely used to evaluate the functional outcome after knee arthroplasty [16]. The AKSS is used to evaluate pain and joint function (score 0 lowest, score 100 highest). WOMAC index is a health status instrument, used to assess everyday fitness (score 10 lowest, score 0 highest). The forms for these two scores instruments were completed by the patients and reviewed by the study nurse.

The LOS was evaluated as part of the study design and counted as postoperative nights in the hospital. Consumption of analgesic drugs was monitored, because the intake of pre- and postoperative medications can influence the outcome. The ‘The Oxford League Table of Analgesic Efficacy’ [37] and an in-house classification of analgesic drugs were used to compare the need for analgesic drugs (Table 2).

**Table 2:** In-house classification of analgesic drugs

Factor 1 Non-opioids NSAID	Factor 2 Non-opioids Others	Factor 3 Opioids Low potential	Factor 4 Opioids High potential	Factor 6 Epidural Application
Acetyl salicylic acid	Paracetamol oral	Paracetamol/codeine	Oxycodone	Clonidine/ropivacaine/sufentanil
Celecoxib	Paracetamol parental	Tramadol	Pethidine	
Diclofenac	Metamizole	Tilidine	Piritramide	
Ibuprofen				
Etoricoxib				

In-house classification of analgesic drugs representing epidural application and newer drugs, based on the Oxford table [37]. The factor indicates the multiplication factor for the administered dose. The sum of the calculated doses represents the total medication. For example: 800 mg ibuprofen factor 1 = 800 mg cumulative; paracetamol 1,000 mg (factor 2) + pethidine 50 mg (factor 4) = 800 + 2,000 + 200 = 3,000 mg cumulative

### Primary evaluation variable

The primary evaluation variable of the study was the ‘area under curve’ (AUC) for AKSS. The AUC was chosen as a suitable parameter, because it provides an integrative description of the patient’s progress, as well as the difference in the progress between the two study groups.

The AUC represents the area under the polygon connecting the mean values of AKSS at each visit. The values were corrected using linear interpolation to allow comparison between groups on the same day. The baseline-corrected AUC was used for the analysis, so as to avoid the possible interference effect of the patient’s preoperative condition on the interpretation of postoperative results.

### Secondary evaluation variables

The secondary evaluation variables were demographic data, co-morbidities, WOMAC index score, LOS, and analgesic drug consumption. The consumption of analgesic medication was monitored by drug, dosage, and day. Descriptive statistics were used for the analysis of the demographic data and co-morbidities. The AKSS values for single visits were also analyzed, as they provided more detailed information about the patient’s progress than the cumulative analysis. WOMAC index score was evaluated, *with* and *without* baseline-corrected AUC, and the LOS compared between the two study groups. Drug consumption was monitored and analyzed in an exploratory manner, using an in-house classification of analgesic drugs (Table 2).

### Statistical methods

Prior to the study, a power calculation was performed. A two times faster improvement within the first week of recovery was assessed as clinically relevant; the corresponding AUC difference should be detected by the trial with alpha = 5 % and a power of 80 %. The amount and variation for AKSS improvement has been estimated from the literature data and own experience. This calculation procedure resulted in 67 patients required per study group, and by adding a 10% anticipated drop out, led to a total of 150 patients for the study.

It was anticipated that the *fast-track group* will achieve better improvement within the first weeks. Nevertheless, it seemed more relevant to look at an overall measure of better or worse recovery integrating the whole time interval from operation to last visit at 3 months. Thus, the primary endpoint of this trial was the integrated value (AUC) of the AKSS within the 3 months after the TKA.

Analyzing data from literature, we generated several scenarios describing typical AKSS to time curves after knee surgery and their variance. All these curves present a steep increase within the first week and a modest further improvement until a final value is reached (at least after about 3 months). An average of AUCs resulting from these scenarios was used as the null hypothesis. The alternative AUC hypothesis was deduced from the same scenario with two time steeper increase in the first week and modest (i.e. linear) further improvement to the same final value. An AUC difference of that size should be detected with alpha = 5 % and power of 80 %.

The randomization was processed by the Center for Clinical Trials (Bremen, Germany) being immediately informed per fax about recruitment and allocation of each patient was transferred to the hospital per fax. Randomization used software RITA 1.05 with Biased Coin Design (BCD). Randomization parameters: *p* = 2/3, random generator Mersenne Twister, seed newly generated (and documented) for each calculation, block = 8.

All statistical analyses were performed using SAS software version 9.1. To examine the difference between the two study groups, *t* test and Wilcoxon test were used for continuous variables, Chi^2^ test and Fisher exact test for categorical. The tests for other evaluation variables were applied for explorative use. Significance level was set to 0.05 without adjustment for multiplicity. Statistical analyses were performed for the intention-to-treat (ITT) and per-protocol (PP) cohorts. ITT analysis was performed, using Last Observation Carried Forward imputation method.

The whole AKSS score (sum of knee and functional score) as primary criterion was used in the analysis. The AKSS (sum of both subscores) was chosen, because it represents the overall criterion for postoperative development and provides the most clinically relevant evidence directly related to the primary objective of the trial. This is in compliance with the ICHE9 guideline, which supports the use of only one primary variable [38].

## Results

### Demographic data

A summary of the patients’ demographic characteristics is shown in Table 1. In both study groups, the distribution of male and female patients, age, BMI, and other physical characteristics were similar. Two patients were treated for Ahlbäck’s disease *(fast-track rehabilitation* group); one patient was treated for posttraumatic disease (*standard rehabilitation* group). All other patients were treated for degenerative arthritis.

### Patient disposition during the study

**Fig. 1:**
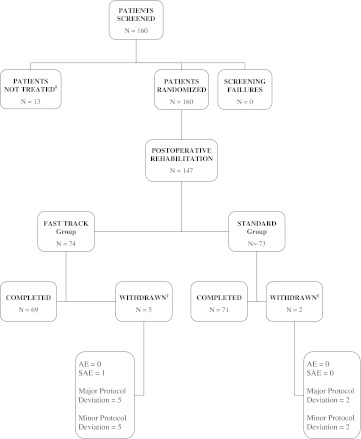
Patient disposition. ^#^Patients were not treated for the following reasons: violations of the inclusion criteria (*N* = 4), patient’s request (*N* = 4), incorrect randomization (*N* = 2), advanced operation (1), stroke before the operation (*N* = 2). ^§^Patients in each group were withdrawn from the study due to both major and minor protocol deviations and in one case also due to one SAE. *AE* adverse event, *SAE* serious adverse event

A total of 160 patients were screened and randomized into the study between 1 January 2006 and 30 June 2007. Of these, 13 patients did not undergo surgery (drop-out or protocol violators) due to violation of the inclusion criteria (*N* = 4), patient’s own request (*N* = 4), false randomization (*N* = 2), advanced operation (*N* = 1), and cerebral infarction (*N* = 2). As shown in Fig. 1, 147 patients were included in the intention-to-treat (ITT) analysis (73 patients in the *standard rehabilitation* group and 74 patients in the *fast-track rehabilitation* group) and 140 patients in the per-protocol (PP) analysis (69 patients in the *standard rehabilitation* and 71 patients in the *fast-track rehabilitation*). The reasons for withdrawal from the ITT analysis are summarized in Fig. 1.

### Area under the time curve for the AKSS

**Fig. 2:**
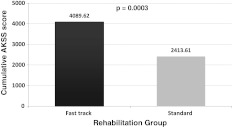
Cumulative AKSS score for patients undergoing TKA (per-protocol cohort). Cumulative AKSS score for patients undergoing a TKA in the per-protocol cohort. The data represents men for the AUC values for *fast-track rehabilitation* group and for the *standard rehabilitation* group. The data show statistically significant difference in favor of the *fast-track rehabilitation* group (*p* = 0.0003, *t* test and Wilcoxon test). *AKSS* American Knee Society Score, *TKA* total knee arthroplasty, *AUC* area under the curve

The baseline-corrected area under the time curve (AUC) for AKSS (shown as mean ± SD) were 4,089.62 ± 2,582.02 (95 % CI 3,469.35–4,709.89) for the *fast-track rehabilitation* group and 2,413.61 ± 2,774.74 (95 % CI 1,756.83–3,070.38), for the *standard rehabilitation* group (Fig. 2). The results of this primary outcome of the study for the PP population cohort were statistically significantly different and in favor of the *fast-track rehabilitation* group versus *standard surgery* group (*p* = 0.0003, *t* test and Wilcoxon test).

**Fig. 3:**
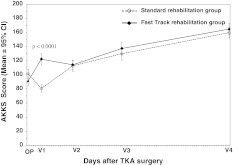
By-visit AKSS score for patients undergoing TKA (per-protocol cohort). Mean and 95 % CI values of American Knee Society Score (AKSS) by visits for patients in the *fast-track rehabilitation* group and the *standard rehabilitation* group (per-protocol cohort). The data show statistically significant difference in favor of the *fast-track rehabilitation* group at visit 1 (*p* < 0.0001,* t* test and Wilcoxon test). *OP* day of surgery. The AKSS is used to evaluate pain and joint function; higher values indicate patients’ better condition (score 0 lowest, score 100 highest). The whole AKSS score (sum of knee and functional score) as primary criterion was used in the analysis. The AKSS (sum of both subscores) was chosen, because it represents an overall criterion for postoperative development and provides the most clinically relevant evidence directly related to the primary objective of the trial

As shown in Fig. 3, on the day of the surgery (OP) the analysis of AKSS showed no statistically different scores between the study groups. However, at visit 1 (V1; day 5–7 after surgery), an increase in the AKSS score was seen when compared with the preoperative values for the *fast-track rehabilitation* group, whereas a decrease was seen for the *standard rehabilitation* control group. The AKSS score difference between the study groups was highly statistically significant at V1 (*p* < 0.0001), i.e., 122.25 versus 80.52 (*t* test and Wilcoxon test). At the subsequent visits, all AKSS scores were numerically higher in the *fast-track rehabilitation* group than in the *standard rehabilitation* group, although these differences were not statistically significant (Fig. 3).

### WOMAC osteoarthritis index

**Fig. 4:**
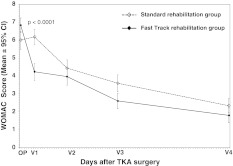
WOMAC osteoarthritis index score by-visit (per-protocol cohort). By visits mean and standard deviation values for WOMAC index score for patients in the *fast-track rehabilitation* group and the *standard rehabilitation* group (per-protocol cohort). *OP* day of surgery. WOMAC index is a health status instrument used to assess everyday fitness; lower values indicate patients’ better condition (score 0 highest, score 10 lowest) [34]

For WOMAC osteoarthritis index, the results of this study showed a similar pattern as for the AKSS. Earlier improvements in the fast-track rehabilitation group are evident, with better values at each visit in comparison with the *standard rehabilitation* group. The results are statistically highly significant WOMAC osteoarthritis index at V1: (4.24 ± 1.94/6.19 ± 1.79; *p* < 0.0001, *t* test and Wilcoxon test), not significant at V2: *p* = 0.1636, highly significant at V3: *p* = 0.0009, and significant at V4: *p* = 0.0123 (Fig. 4). The baseline-corrected AUC for WOMAC index was significantly in favor of the *fast-track rehabilitation* group (*p* = 0.0015, PP cohort; *p* = 0.0020, ITT cohort) (Table 3).

**Table 3:** AUC WOMAC index, baseline corrected (per-protocol cohort and intention-to-treat cohort)

Population	Postoperative rehabilitation	*N*	Median	95 % CI		*t* test *p* value	Wilcoxon *p* value
PP	Fast-track	69	271.47	240.24	302.70	0.0015	0.0022
	Standard	71	345.42	312.25	378.59		
ITT	Fast-track	74	275.50	245.33	305.67	0.0020	0.0028
	Standard	73	345.74	313.16	378.32		

Comparison of the WOMAC, a health status instrument used to assess everyday fitness [35], in patients in the *fast-track rehabilitation* group and *standard rehabilitation* group

*CI* confidence interval, *ITT* intention-to treat, *N* number of patients, *PP* per-protocol, *WOMAC* Western Ontario and McMaster Universities Osteoarthritis Index

### Drug consumption

**Fig. 5:**
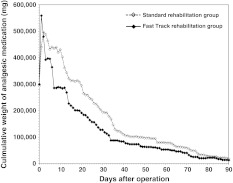
Weighted cumulative consumption of analgesic medications (intention-to-treat cohort). Weighed cumulative intake of analgesic medication (in mg), according to the in-house classification (Table 2) for patients in the *fast-track rehabilitation* group and the *standard rehabilitation* group. Except for the first 2 days after the surgery, patients in the *fast-track rehabilitation* group needed a significantly lower amount of analgesic drugs than patients in the *standard rehabilitation* group (Wilcoxon test *p* = 0.0019; ITT cohort)

The weighted cumulative need for analgesic drugs in the *fast-track rehabilitation* group was higher in the first 2 days; thereafter, the need for analgesia was lower than in the *standard rehabilitation* group (Fig. 5). The total sum of analgesic drugs used per patient over 91 days was systematically lower in the *fast-track rehabilitation* group (Kolmogorov–Smirnov two-sample test: *p* = 0.0282, Wilcoxon test: *p* = 0.0019).

**Fig. 6:**
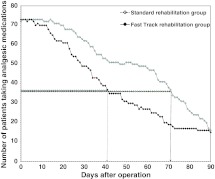
Patients receiving analgesic medications (intention-to-treat cohort). Number of patients using analgesic drugs for patients in the *fast-track rehabilitation* group and the *standard rehabilitation* group (ITT cohort). The* horizontal* *arrow lines* indicate 50 % (half of the patients) in each study group and the *vertical arrow lines* indicate the postoperative day on which half of the patients stopped consuming analgesic drugs. Thus, 50 % of patients in the *fast-track rehabilitation* group needed 30 days less than in the *standard rehabilitation* group to stop consuming analgesic drugs (i.e., 71 days after operation *standard rehabilitation* − 41 days after operation *fast-track rehabilitation* = 30 days difference)

Monitoring of the absolute number of patients with any analgesic medication showed that in the *fast-track rehabilitation* group*,* 50 % of the patients had stopped their analgesic medication after 41 days, whereas in the *standard rehabilitation* group this occurred after 71 days, implying 30 days less on analgesic drugs for patients in the *fast-track rehabilitation* group (Fig. 6).

### Length of stay

**Fig. 7:**
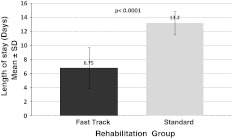
Length of stay in hospital after TKA (per-protocol cohort). Mean and standard deviation values for length of stay (LOS) in the hospital for patients showing a significantly shorter LOS in the *fast-track rehabilitation* group than in the *standard rehabilitation* group (*p* < 0.0001, *t* test and Wilcoxon test, per-protocol cohort)

The LOS in the orthopedic unit of the hospital was 6.75 days for patients in the *fast-track rehabilitation* group. This result is significantly shorter (*p* < 0001) than 13.20 days observed for patients in the *standard rehabilitation* group (Fig. 7).

### Safety

Overall, 23 AEs were observed for 147 patients. There were 20 AEs assessed as related to the procedure and 3 AEs [cerebral stroke (*N* = 1), viral infection (*N* = 1), and renal colic (*N* = 1)], assessed by the investigator as not related to the procedure.

The intensity of the procedure-related AEs was assessed as severe (*N* = 2) and minor (*N* = 18). The two severe AEs were: deep infection (*fast-track rehabilitation* group) and humerus fracture (*standard rehabilitation* group). Minor AEs (*N* = 7 in the *fast-track rehabilitation* group, *N* = 11 in the *standard rehabilitation* group) were: stiffness (*N* = 13), urinary tract infection (*N* = 2), subluxations of the patella (*N* = 2), tibial fissure (*N* = 1). None of the patients died during the study.

## Discussion

Clinical pathway treatment in TKA is recognized as a team-approach tool that can achieve better medical outcome and economic performance than standard care, while minimizing complications and optimizing patient-centered care [23, 24, 27, 39, 40]. The aim of the present prospective, randomized, and controlled study was to evaluate the feasibility of implementing a comprehensive *fast-track* rehabilitation concept in Germany and its effects on the recovery pattern after TKA, using the pathway-controlled *fast-track rehabilitation* as compared with the *standard* *rehabilitation*.

The results of our study show that after a TKA, the *fast-track rehabilitation* group reached the AKSS (the primary variable of the study), with a significantly higher *cumulative* AUC score (4,089.62) than in the *standard rehabilitation* group (2,413.61) (*p* = 0.0003, *t* test and Wilcoxon test) (Fig. 2). Similarly, when analyzing the data *by visit*, a higher AKSS was reached for the *fast-track rehabilitation* group already at visit 1 (day 5–7 after surgery), whereas for the *standard rehabilitation* group at this visit, a decrease in AKSS was seen, with a statistically significantly lower values, i.e., 122.25 versus 80.52 (*p* < 0.0001, *t* test and Wilcoxon test) (Fig. 3). Here it is essential to keep in mind that at visit 1 (day 5–7 after surgery), all patients in the *fast-track rehabilitation* group left the specialist orthopedic clinic, whereas the patients in the *standard rehabilitation* group remained in the orthopedic clinic for a significantly longer time. The average LOS at the orthopedic clinic for patient in the *fast-track rehabilitation* group was 6.75 days, which was significantly shorter than 13.2 days for patients in the *standard rehabilitation* group (*p* < 0001).

After the discharge from the orthopedic clinic, all patients were transferred to a rehabilitation center in which the concept of *fast-track* rehabilitation did *not* continue. This is reflected in the values of the AKSS at visits V2, V3, and V4, which did not show a statistically significant difference between the two rehabilitation groups, although the AKSS values were all numerically higher for the *fast-track rehabilitation* group than for the *standard rehabilitation* group (Fig. 3). Our results of the LOS, together with the data of the AKSS at V1 clearly show that the main effect of the *fast-track rehabilitation* group recovery process occurred during the first days after the TKA. An open question remains whether a further improvement would have occurred if the patients of the *fast-track rehabilitation* group would have been maintained on a post-discharge therapy that incorporates the *fast-track rehabilitation* concept. Indeed, this could be seen both as one of the limitations of the current study design as well as a future challenge to orthopedic units and rehabilitation centers that deal with patients undergoing TKA.

The AKSS is a widely used outcome in TKA, particularly in the USA [41], and is a highly suitable instrument to allow comparison with other published studies. The AKSS focuses on joint functions such as range of motion of the joint and perception of pain. However, the parameters of *‘everyday function’* are not fully covered by the AKSS and are better represented through the WOMAC index. The WOMAC index reflects health status and assesses everyday fitness, including social activity of patients with osteoarthritis of the hip or knee using 24 parameters [35, 36, 42]. For this reason, information was also collected for the WOMAC index as a secondary parameter of the present study. The results of the WOMAC index showed that throughout the 3-month observation period, the *fast-track rehabilitation* had long lasting positive effects on the patients and furthermore, that the results of the WOMAC index emphasize the reliability and consistency of the AKSS.

The perception of pain level plays an important role for both AKSS and WOMAC index. The outcomes for both scores therefore might be biased by different medications used to control pain (e.g., higher dosage of analgesic drugs in the *fast-track rehabilitation* group). To exclude such a potential bias, the time of analgesic drug intake, frequency, and amount of the analgesic drug were monitored and analyzed in our study. The validated Oxford league of pain table was not appropriate for our study because it does not include the standard epidural application of clonidine/ropivacaine/sufentanil, which was administered for patient-controlled anesthesia within the first 24 h postoperatively. Therefore, we used an in-house, pharmacologist-approved, classification of the analgesic drugs (Table 2). Although this classification is not validated, the results from the present study indicate that excessive drug consumption in the *fast-track rehabilitation* group did not occur. The use of more analgesics in the *fast-track* rehabilitation group for the first 2 days after TKA may reflect the fact that these patients were mobilizing on the day of the surgery as opposed to the patients in the *standard* *rehabilitation* group who were mobilized on the second day after surgery. After the initial increase, the subsequent consumption of medication (in mg) was considerably lower for the *fast-track rehabilitation* group throughout the rest of the observation period than for the *standard* *rehabilitation* group (Fig. 5). Additionally, the 50 % end point (half of the patients in each study group) of analgesic drug consumption occurred about 30 days earlier in the *fast-track rehabilitation* group than in the *standard rehabilitation* group (Fig. 6). Such a result reduces the possible bias of excessive analgesic drug consumption even further. Nonetheless, the lack of a standardized pain management is a methodological weakness of the study. When the study was initiated, the awareness of in-house classification pain management system was not as far developed as it is now.

This study shows data on the feasibility of *fast-track rehabilitation* after TKA in an orthopedic hospital unit in Germany. Conclusions drawn from our data are similar to other studies such as those performed in Denmark, where the concept of fast-track surgery in TKA has been widely implemented for more than 10 years [11]. Our data show that early mobilization after the surgery with intensive early physiotherapy has a sustainable effect on the midterm outcome (3–12 months). This finding is also in agreement with the recent data of a clinical study reported by Larsen et al. [43], who concluded that there is a need for an additional postoperative rehabilitation after fast-track total knee arthroplasty and unicompartmental knee arthroplasty regarding early functional outcome; those patients who experienced no or only mild pain and who had good functional abilities at 4 months were associated with high health-related quality-of-life and patient satisfaction at 4- and 12-month follow-up. Similar results have been found in total hip replacement [44]. Positive findings have also been reported recently with patients who had to undergo revision TKA (due to non-septic reasons). The results indicate that even such patients may be included in fast-track protocols and furthermore, underline the usefulness of the fast-track surgery and rehabilitation concepts even in less standardized procedures, which typically have more extensive surgical trauma that leads to a corresponding increase in the surgical stress responses [45].

In the present study, the target for discharge after TKA was 6 days, which was in compliance with the German DRG system in 2005 [15]. At that time, this implied a reduction in LOS of almost 50 %, as compared with the average LOS in Germany of 14.1–14.3 days [7, 8]. The data from our study show that using the *fast-track rehabilitation* concept, a high AKSS together with a reduction in LOS, and fewer AEs were safely achievable; the patients’ daily living parameters, as assessed by the WOMAC index score, were favorable and statistically highly significant. Furthermore, the *fast-track rehabilitation* concept used in the present study is patient-focused, is feasible, transferable to other centers, and may have economic implications through reduced hospital costs and health-care benefit contributions. Indeed, the results of our study endorse and contribute some answers to the recently posed question “*Why still in hospital after fast-track hip and knee arthroplasty?*” [46].
